# High-Glucose-Induced Rab20 Upregulation Disrupts Gap Junction Intercellular Communication and Promotes Apoptosis in Retinal Endothelial and Müller Cells: Implications for Diabetic Retinopathy

**DOI:** 10.3390/jcm9113710

**Published:** 2020-11-19

**Authors:** Dongjoon Kim, Casey Stottrup Lewis, Vijay P. Sarthy, Sayon Roy

**Affiliations:** 1 Department of Medicine, Boston University School of Medicine, Boston, MA 02118, USA; djkim@bu.edu (D.K.); cstottrup@bu.edu (C.S.L.); 2 Department of Ophthalmology, Boston University School of Medicine, Boston, MA 02118, USA; 3 Department of Ophthalmology, Northwestern University Feinberg School of Medicine, Chicago, IL 60611, USA; vjsarthy@northwestern.edu

**Keywords:** Rab20, connexin 43, apoptosis, gap junctions, diabetic retinopathy

## Abstract

To investigate whether high glucose (HG) alters Rab20 expression and compromises gap junction intercellular communication (GJIC) and cell survival, retinal cells were studied for altered intracellular trafficking of connexin 43 (Cx43). Retinal endothelial cells (RRECs) and retinal Müller cells (rMCs) were grown in normal (N; 5 mM glucose) or HG (30 mM glucose) medium for seven days. In parallel, cells grown in HG medium were transfected with either Rab20 siRNA or scrambled siRNA as a control. Rab20 and Cx43 expression and their localization and distribution were assessed using Western Blot and immunostaining, respectively. Changes in GJIC activity were assessed using scrape load dye transfer, and apoptosis was identified using differential dye staining assay. In RRECs or rMCs grown in HG medium, Rab20 expression was significantly increased concomitant with a decreased number of Cx43 plaques. Importantly, a significant increase in the number of Cx43 plaques and GJIC activity was observed in cells transfected with Rab20 siRNA. Additionally, Rab20 downregulation inhibited HG-induced apoptosis in RRECs and rMCs. Results indicate HG-mediated Rab20 upregulation decreases Cx43 localization at the cell surface, resulting in compromised GJIC activity. Reducing Rab20 expression could be a useful strategy in preventing HG-induced vascular and Müller cell death associated with diabetic retinopathy.

## 1. Introduction

Diabetic retinopathy is the leading cause of blindness in the working-age population in Western countries [1]. The early stages of this devastating disease are characterized by high-glucose (HG)-mediated microvascular changes [2,3,4] as well as glial changes [5,6,7,8,9] in the retina, leading to loss of retinal endothelial cells, pericytes, and Müller glial cells. Several studies suggest that hyperglycemia-induced disruption of gap junction intercellular communication (GJIC) plays a critical role in the pathogenesis of diabetic retinopathy [10,11,12,13,14,15,16,17,18,19,20]. It is also important to note that HG is known to compromise other cellular junctions, including tight junctions, in retinal cells [21,22]. With respect to gap junctions, studies indicate that translocation and modification of connexins are critical in regulating GJIC activity [23,24], and Rab20, a small GTPase, may impact the intracellular trafficking of connexin 43 (Cx43) [25]. However, it is currently unknown whether intracellular trafficking of Cx43 is altered by HG via regulation of Rab20 and whether such changes influence cell survival in the context of diabetic retinopathy.

GJIC enables the exchange of small molecules through gap junction channels granting passage of ions, nutrients, and other signaling molecules (up to 1 kD) between neighboring cells and is essential for the maintenance of retinal homeostasis [26]. Connexin monomers oligomerized into hexameric proteins are transported to the plasma membrane, where they function as active gap junction channels. In particular, connexin 43 (Cx43) gap junction channels are abundantly expressed in the retina [27] and participate in the regulation of the blood–retinal–barrier as well as maintenance of retinal vascular and glial homeostasis [16,28]. Retinal vascular cells exchange various ions and small metabolites including cyclic AMP (cAMP), Ca^2+^, and other molecules among them essential for cell survival, growth, proliferation, and homeostasis [29,30]. Importantly, our previous studies have indicated that HG or diabetes downregulates Cx43 expression in retinal vascular cells, compromising GJIC, thereby triggering apoptosis [13,14,17,18,31,32]. Interestingly, downregulating Cx43 alone in rats using a siRNA strategy resulted in accelerated retinal vascular cell death and vascular leakage associated with diabetic retinopathy [33]. A clinical study showed that retinas of patients with diabetic retinopathy exhibit significantly reduced Cx43 expression, which was associated with increased retinal vascular cell loss, highlighting the relevance of Cx43 downregulation in human diabetic retinopathy [20].

Studies indicate that Müller glial cells are in close apposition with each other, making frequent contact with the capillaries in the retina [34]. Cx43 are abundantly present in the apical processes of Müller cells located at the outer limiting membrane [35]. Of note, Cx43 has been established as the major gap junction protein between Müller cells in lower vertebrates [36]. Cx43 immunoreactivity was detected in Müller cells in human retinas [37]. Taken together, these studies indicate that Cx43 plays a central role in intercellular communication and helps regulate cell survival in retinal Müller cells [38]. Conversely, findings from one of our recent studies indicate that HG plays a deleterious role in retinal Müller cells by downregulating Cx43 expression, compromising GJIC, and promoting apoptosis [16]. The literature supports the implication that maintenance of cell–cell communication is essential for retinal endothelial cell and Müller cell survival.

Migration of Cx43 gap junction proteins to the cell surface is essential for GJIC activity. GTPases play an important role in regulating intracellular trafficking and facilitating membrane fusion and transport of proteins to the cell surface. Intracellular trafficking pathways comprise overlapping relays of GTPases acting sequentially, coordinating transport and membrane fusion by cycling between the membrane-bound, active GTP, and inactive, cytosolic GDP-states. One GTPase, Rab20, has been identified as a potential regulator of Cx43 trafficking [25]. Although the exact function of Rab20 is not fully understood, early data suggests it may hinder trafficking of Cx43 from the endoplasmic reticulum to the Golgi apparatus [25]. Rab20 assumes a perinuclear localization, presumably at the Golgi, and has been shown to reduce Cx43 localization at the cell surface [25].

Although much has been elucidated in the deleterious effects of HG on GJIC activity and retinal cell apoptosis, the exact mechanism by which intracellular transport of Cx43 is reduced remains unclear. GTPases likely play a critical part in this pathway and warrant further exploration. Therefore, the current study was undertaken to investigate whether HG alters Rab20 expression and subsequently affects Cx43 localization, GJIC activity, and cell survival in RRECs and rMC-1.

## 2. Materials and Methods

### 2.1. Cell Culture

Endothelial cells exhibiting von Willebrand factor (vWF) were isolated from rat retinal capillaries (RRECs) as previously described [39] and used in the present study. Rat retinal Müller cells (rMC-1) were previously characterized as Müller cells based on long and slender shape morphology and expression of cellular retinaldehyde-binding (CRALBP) protein [40]. To determine the downstream effects of HG on Rab20 expression, cultures of RRECs or rMC-1 were grown for 7 days in 35 mm Petri dishes at 37 °C in normal (N; 5 mM D-glucose) or high-glucose (HG; 30 mM D-glucose) Dulbecco’s modified Eagle’s medium (DMEM) containing 10% fetal bovine serum (Sigma, St. Louis, MO, USA), antimycotics, and antibiotics. RRECs were plated at a density of 10,000 cells for normal glucose and at 12,000 cells for HG conditions to reach confluence after 7 days. In parallel, rMC-1 were plated at a density of 7500 cells for normal glucose and at 9000 cells for HG conditions to reach confluence after 7 days.

### 2.2. Transfection with Rab20 siRNA

To assess whether reducing Rab20 overexpression influences Cx43 localization, GJIC activity, and cell viability, RREC or rMC-1 grown under HG condition were subjected to transfection with 40 nM Rab20 siRNA (Qiagen, Germantown, MD, USA) or scrambled siRNA (scram; Ambion, Austin, TX, USA) as a negative control using 8 μM Lipofectin (Invitrogen, Grand Island, NY, USA) diluted with Opti-MEM reduced serum medium (Invitrogen). Transfected cells were harvested and subjected to scrape load dye transfer (SLDT) analysis, immunostaining, immunoprecipitation, Western blot (WB) analysis, or differential dye staining after 7 days of HG exposure.

### 2.3. Immunostaining

To determine whether changes in Rab20 levels alter Cx43 distribution and localization, coimmunostaining was performed. Briefly, RRECs or rMC-1 were plated on glass coverslips and subjected to 4% paraformaldehyde fixation for 15 min at room temperature, then exposed to ice-cold methanol and incubated with 2% bovine serum albumin (BSA) for 60 min to block nonspecific antibody binding. Incubation with rabbit-Cx43 antibody solution (1:100; Cell Signaling, Danvers, MA) and mouse-Rab20 antibody solution (1:100; Abcam, Cambridge, MA, USA) occurred overnight in a moisture chamber at 4 °C. The next day, the coverslips were incubated with antirabbit secondary antibody conjugated with FITC (1:200; Jackson ImmunoResearch Laboratories, West Grove, PA, USA) and antimouse secondary antibody conjugated with Rhodamine Red (Jackson ImmunoResearch Laboratories) for 1 h at room temperature in the dark. Cells were photographed under a confocal microscope (LSM710; Zeiss, Göttingen, Germany), and Cx43/Rab20 immunofluorescence staining data were obtained by counting Cx43 plaques on adjacent cell bodies and analyzing Rab20 immunofluorescence values normalized by the total number of cells per field using NIH ImageJ software, respectively. Specifically, Cx43 punctate “dots” in adjacent cells were counted in at least ten random fields from each experimental group, which were then normalized by the total number of cells per field to quantify Cx43 immunostaining [18].

### 2.4. Immunoprecipitation (IP) and WB Analysis

Total protein was isolated from RRECs or rMC-1, and IP for Rab20 was performed with the use of agarose beads as previously described [19]. A total of 250 μg of total protein from the experimental groups, as determined by the bicinchoninic acid protein assay, was immunoprecipitated for Rab20 and were loaded into each lane on a 10% SDS-polyacrylamide gel. WB procedure was performed as described previously [19]. Following semidry transfer, PVDF membranes were blocked for 1 h with 5% nonfat dry milk dissolved in TTBS. After blocking, the membranes underwent several washes with TTBS and were exposed to an antigoat Rab20 antibody (1:500; Santa Cruz Biotechnology, Santa Cruz, CA, USA) and incubated overnight at 4 °C. The following day, membranes were subjected to washes with TTBS and exposed to alkaline-phosphatase conjugated antigoat IgG (1:5000; Santa Cruz Biotechnology) as secondary antibody. Membranes were then subjected to a chemiluminescent substrate (Immun-Star; Bio-Rad, Hercules, CA, USA) and developed using a digital imager (Fujifilm LAS-4000). Densitometric analysis of the signals was performed using the NIH ImageJ software.

### 2.5. Differential Dye Staining

To investigate the effects of altered Rab20 expression on cell viability, differential dye staining assay was performed to identify apoptotic cells [41]. The principle of the differential dye stain assay takes advantage of the gradual loss of cell membrane properties during apoptosis. When cells undergo apoptosis, they are characterized by loss of cell membrane integrity, which allows entry of ethidium bromide and acridine orange into the cells. Acridine orange can enter both viable and apoptotic cells and intercalate with the DNA, resulting in green fluorescence, whereas ethidium bromide can enter only the apoptotic cells due to their compromised membrane integrity, allowing mixing of the two fluorescent dyes [41]. This mixing produces a variety of colors ranging from yellow/light orange fluorescence, signifying early-stage apoptosis, and dark orange/red fluorescence, signifying late-stage apoptosis. At the time of harvest, RRECs or rMC-1 cultured on coverslips were washed with PBS several times and exposed to a mixture of ethidium bromide (25 μg/mL) and acridine orange (25 μg/mL) for 10 min at room temperature. The cells were then subjected to PBS washes and mounted onto a glass slide using a SlowFade Diamond Antifade mountant. Apoptotic cells were identified using a DAPI filter in at least ten random fields and imaged through a digital camera using a fluorescence microscope (Nikon TE2000-S). The total number of cells in each field was identified using the NIH ImageJ software. This was performed by subtracting background fluorescence, adjusting threshold values, and analyzing particles. Subsequently, analysis of apoptotic cells was performed by assessing the apoptotic index, which is the number of cells undergoing apoptosis divided by the total number of cells per field expressed as a percentage.

### 2.6. Scrape Load Dye Transfer (SLDT)

SLDT assay is a technique to assess cell–cell coupling. Cells grown to confluent monolayer are subjected to random cuts allowing small molecules <1 kD in size to transverse between cells through gap junctions [42,43]. In particular, a tracer dye, Lucifer Yellow (MW 457), can pass between contiguous cells from the point of “cut” through gap junctions. The number of cells that the Lucifer Yellow dye traverses perpendicular to the “cut” represents the number of dye-coupled cell layers. Therefore, cells exhibiting a greater extent of dye coupling represent increased Cx43-mediated GJIC. Briefly, RRECs or rMC-1 grown on coverslips were washed with PBS containing 0.01% Ca^2+^ and Mg^2+^ several times. Random cuts were then made in the monolayer using a razor blade. A solution containing PBS with 0.05% Lucifer Yellow (LY; Molecular Probes) was applied to the cells and incubated at room temperature for 5 min. Following incubation, cells were rinsed with PBS containing 0.01% Ca2+ and Mg2+ three times. Cells were then fixed with 4% paraformaldehyde, visualized using a FITC filter, and photographed using a fluorescence microscope (Nikon). To evaluate GJIC activity, dye-coupled cell layers were counted and analyzed in at least ten random fields.

### 2.7. Statistical Analysis

Data are expressed as means ± SD. One-way ANOVA followed by Bonferroni post-hoc test was performed to assess differences between multiple groups. Six replicates were performed for each experiment, and the data were analyzed for statistical significance. *p* < 0.05 represented statistical significance.

## 3. Results

### 3.1. High Glucose Upregulates Rab20 Protein Expression in RRECs and rMC-1

**Figure 1:**
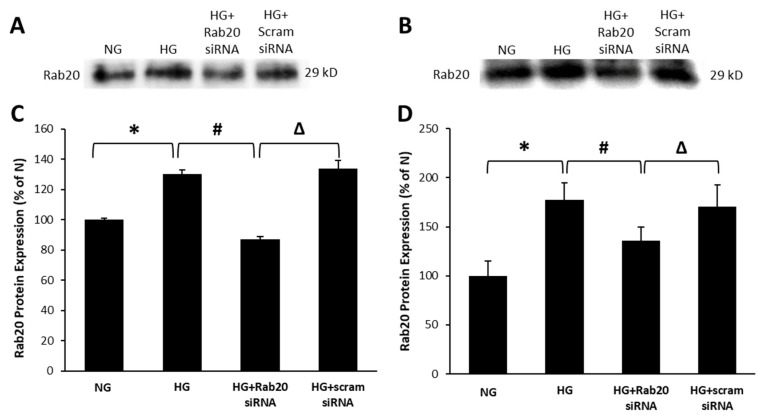
Effects of high glucose (HG) and Rab20 siRNA on Rab20 expression in retinal endothelial cells (RRECs) and rMC-1. Representative images of WB using immunoprecipitated Rab20 protein shows HG significantly upregulates Rab20 expression in (**A**) RRECs and (**B**) rat retinal Müller cells (rMC-1). Graphical illustration of cumulative WB data indicates that cells grown in HG and transfected with Rab20 siRNA exhibit significantly reduced Rab20 expression compared to that of cells grown in HG alone in both (**C**) RRECs and (**D**) rMC-1. Data are expressed as mean ± SD. * *p* < 0.05, *n* = 6; # *p* < 0.05, *n* = 6; Δ *p* < 0.05, *n* = 6.

Following immunoprecipitation for Rab20, the expression level for Rab20 was significantly elevated in RRECs or rMC-1 grown under HG condition compared to those grown in normal glucose (NG) condition (RRECs: 130 ± 3% of NG vs. 100 ± 1% of NG; *p* < 0.05; *n* = 6; Figure 1; rMC-1: 177 ± 17% of NG vs. 100 ± 15% of NG; *p* < 0.05; *n* = 6; Figure 1). As expected, Rab20 levels were downregulated in cells grown under HG condition and transfected with Rab20 siRNA compared to those grown in HG alone (RRECs: 87 ± 2% of NG vs. 130 ± 3% of NG; *p* < 0.05; *n* = 6; Figure 1; rMC-1: 136 ± 14% of NG vs. 177 ± 17% of NG; *p* < 0.05; *n* = 6; Figure 1). There was no significant difference in Rab20 expression between cells grown in HG medium and transfected with scram siRNA and cells grown in HG medium alone. 

### 3.2. Effect of HG and Rab20 Downregulation on Cx43 Distribution and Localization in RRECs and rMC-1

**Figure 2:**
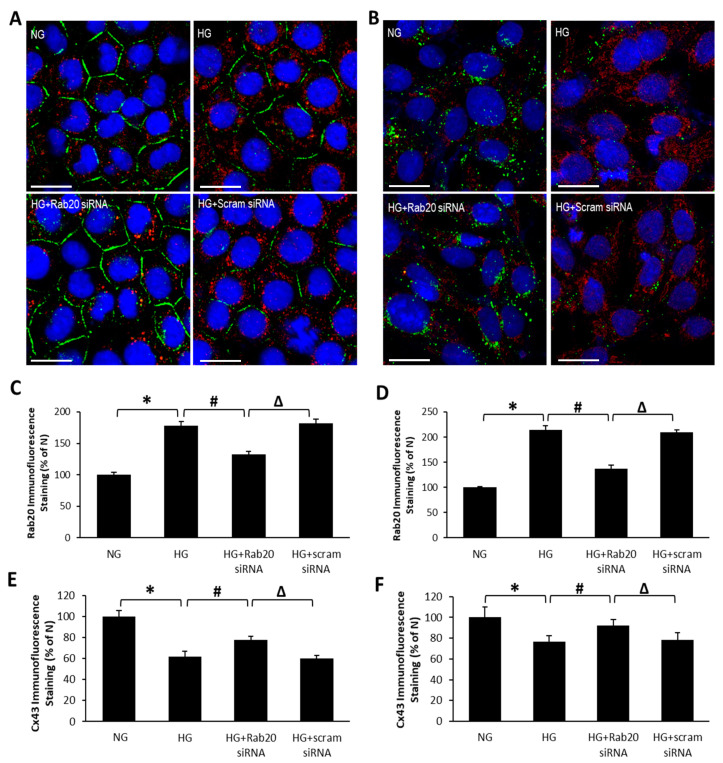
Rab20 siRNA attenuates HG-induced downregulation of connexin 43 (Cx43) expression. Representative images show decreased Cx43 immunoreactivity (green) and increased Rab20 immunoreactivity (red) in (**A**) RRECs and (**B**) rMC-1. Scale bar = 25 μm. Cells transfected with Rab20 siRNA restores Cx43 level. Graphical illustration of cumulative immunofluorescence data shows HG increases Rab20 immunostaining in (**C**) RRECs and (**D**) rMC-1, and that Rab20 siRNA prevents HG-induced decrease in Cx43 plaques in (**E**) RRECs and (**F**) rMC-1. Data are expressed as mean ± SD. * *p* < 0.05, *n* = 6; # *p* < 0.05, *n* = 6; Δ *p* < 0.05, *n* = 6.

To investigate whether Rab20 upregulation influences Cx43 distribution and localization in RRECs or rMC-1, Cx43 immunostaining was performed. As expected, RRECs or rMC-1 grown in HG medium exhibited reduced Cx43 immunostaining (RRECs: 62 ± 3% of NG vs. 100 ± 5% of NG; *p* < 0.05; *n* = 6; Figure 2; rMC-1: 77 ± 6% of NG vs. 100 ± 8% of NG; *p* < 0.05; *n* = 6; Figure 2) compared to cells grown in NG condition. Importantly, when HG-induced Rab20 overexpression was reduced using Rab20 siRNA, a significant increase in Cx43 immunostaining was observed at the cell surface (RRECs: 77 ± 4% of NG vs. 62 ± 3% of NG; *p* < 0.05; *n* = 6; Figure 2; rMC-1: 92 ± 6% of NG vs. 77 ± 6% of NG; *p* < 0.05; *n* = 6; Figure 2). No significant difference in Cx43 immunostaining was observed between cells grown in HG and cells grown in HG transfected with scrambled siRNA. In contrast, Rab20 immunostaining in RRECs and rMC-1 was significantly increased in cells grown in HG medium (RRECs: 178 ± 7% of NG vs. 100 ± 4% of NG; *p* < 0.05; *n* = 6; Figure 2; rMC-1: 214 ± 9% of NG vs. 100 ± 2% of NG; *p* < 0.05; *n* = 6; Figure 2); however, in the presence of Rab20 siRNA, Rab20 immunostaining was significantly decreased (RRECs: 132 ± 5% of NG vs. 178 ± 7% of NG; *p* < 0.05; *n* = 6; Figure 2; rMC-1: 137 ± 7% of NG vs. 214 ± 9% of NG; *p* < 0.05; *n* = 6; Figure 2) whereas scrambled siRNA showed no effects.

### 3.3. Rab20 Downregulation Restores GJIC Activity in RRECs and rMC-1

**Figure 3:**
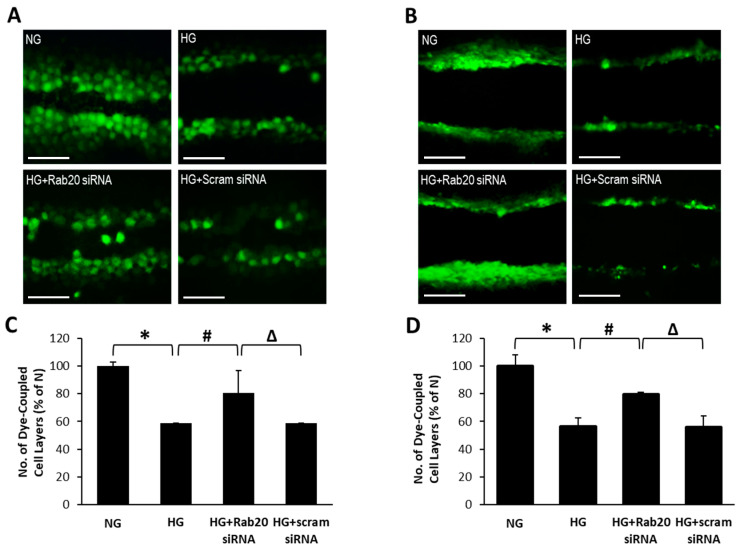
Rab20 downregulation lessens HG-induced decrease in gap junction intercellular communication (GJIC) in RRECs and rMC-1. Representative scrape load dye transfer (SLDT) images show (**A**) RRECs or (**B**) rMC-1 grown in HG exhibit a decrease in the number of dye-coupled cell layers. Scale bar = 100 μm. Graphical illustration of cumulative SLDT data shows siRNA-mediated Rab20 downregulation improves GJIC activity in (**C**) RRECs (* *p* < 0.01, *n* = 6; # *p* < 0.05, *n* = 6; Δ *p* < 0.05, *n* = 6) and (**D**) rMC-1 (* *p* < 0.05, *n* = 6; # *p* < 0.05, *n* = 6; Δ *p* < 0.05, *n* = 6) grown in HG condition. Data are expressed as mean ± SD.

To better understand the association between increased Rab20 expression and GJIC activity, gap-junction-mediated dye coupling between cells was evaluated through the SLDT assay in the context of Rab20 levels. The number of cells that the Lucifer Yellow dye has traversed from the point of “cut” represents the number of dye-coupled cell layers. In RRECs and rMC-1 grown under HG condition, the number of dye-coupled cell layers was significantly reduced compared to those grown in NG condition (RRECs: 59 ± 1% of NG vs. 100 ± 3% of NG; *p* < 0.05; *n* = 6; Figure 3; rMC-1: 56 ± 22% of NG vs. 100 ± 31% of NG; *p* < 0.05; *n* = 6; Figure 3). Importantly, when HG-induced Rab20 upregulation was reduced using Rab20 siRNA, a significant increase in the number of dye-coupled cell layers was observed (RRECs: 81 ± 16% of NG vs. 59 ± 1% of NG; *p* < 0.05; *n* = 6; Figure 3; rMC-1: 79 ± 20% of NG vs. 56 ± 29% of NG; *p* < 0.05; *n* = 6; Figure 3). In parallel, there was no significant difference in the number of dye-coupled cell layers between cells grown in HG and cells grown in HG transfected with scrambled siRNA.

### 3.4. Effect of Rab20 Downregulation on RREC and rMC-1 Cell Survival

**Figure 4:**
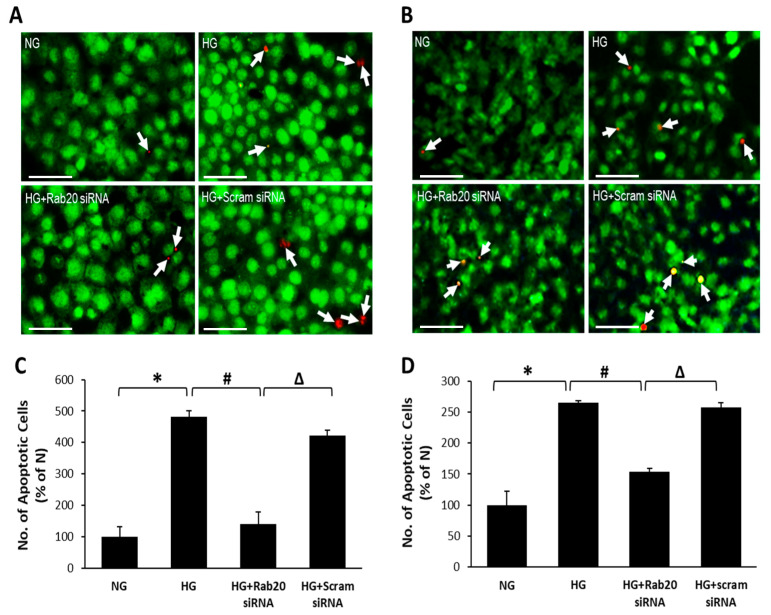
Inhibition of HG-induced Rab20 overexpression protects RRECs and rMC-1 from HG-induced apoptosis. Differential staining assay shows an increased number of apoptotic cells under HG condition, which was abrogated by Rab20 downregulation in (**A**) RRECs and (**B**) rMC-1. Representative images of cells undergoing apoptosis (white arrows). Scale bar = 50 μm. Graphical illustrations of cumulative data indicate that downregulation of Rab20 expression rescues (**C**) RRECs (* *p* < 0.01, *n* = 6; # *p* < 0.05, *n* = 6; Δ *p* < 0.05, *n* = 6) and (**D**) rMC-1 (* *p* < 0.05, *n* = 6; # *p* < 0.05, *n* = 6; Δ *p* < 0.05, *n* = 6) from HG-induced apoptosis. Data are expressed as mean ± SD.

Differential dye staining assay was performed to investigate whether changes in Rab20 expression impact cell survival. As expected, a significant increase in the number of apoptotic cells was observed in RRECs and rMC-1 grown under HG condition compared to cells grown in NG condition (RRECs: 483 ± 18% of NG vs. 100 ± 32% of NG; *p* < 0.01, *n* = 6; Figure 4; rMC-1: 265 ± 3% of NG vs. 100 ± 22% of NG, *p* < 0.05; *n* = 6; Figure 4). Of note, RRECs or rMC-1 grown under HG condition and transfected with Rab20 siRNA showed a significant reduction in the number of apoptotic cells (RRECs: 140 ± 39% of NG vs. 483 ± 18% of NG, *p* < 0.01; *n* = 6; Figure 4; rMC-1: 153 ± 5% of NG vs. 265 ± 3% of NG, *p* < 0.05; *n* = 6; Figure 4). Cells grown in HG medium and transfected with scrambled siRNA showed no significant difference in the number of apoptotic cells compared to those grown in HG medium alone.

## 4. Discussion

To the best of our knowledge, this is the first study demonstrating that HG upregulates Rab20 expression in retinal endothelial cells and Müller cells. Moreover, this aberrant Rab20 upregulation effectively inhibits Cx43 intracellular trafficking to the cell surface, thereby compromising GJIC activity and promoting loss of retinal endothelial cells and retinal Müller cells associated with diabetic retinopathy. Interestingly, when HG-mediated Rab20 upregulation was reduced using Rab20 siRNA, it facilitated Cx43 localization at the cell surface, which is indicative of improved Cx43 intracellular trafficking. The increase in Cx43 localization at the cell surface was concomitant with effective GJIC activity and cell survival. Overall, these results indicate that a HG-induced increase in Rab20 levels may interfere with Cx43 intracellular trafficking and compromise cell–cell communication in retinal endothelial cells and Müller cells.

Although the role of Rab20 in mediating Cx43 intracellular trafficking was examined in the current study, the exact functions of Rab20 are not well understood. Rab20 has been found to regulate phagosome maturation and contribute to the macropinocytic pathway [44,45]. Providing further insight, another study showed that Rab20 expression is upregulated by hypoxia-inducible factor (HIF-1), suggesting that Rab20 may participate in hypoxia-induced apoptosis [46]. Rab20 has also been found to colocalize with the mitochondria in primary tubular cells and other human cell lines [46]. Another recent study revealed that overexpression of Rab20 hindered neurite outgrowth through a hitherto unknown mechanism [47], suggesting that excess Rab20 levels can promote cell death. An additional study reported that Rab20 regulates insulin-stimulated glucose uptake in human and mouse skeletal muscle by facilitating GLUT-4 translocation to the cell membrane [48]. Although these studies highlight the multifaceted functions of Rab20, further studies are needed to better understand how Rab20 influences Cx43 intracellular trafficking.

Findings from this study indicate that HG-mediated Rab20 upregulation in retinal endothelial cells and Müller cells impedes Cx43 localization to the cell surface, and that inhibiting Rab20 overexpression using a siRNA strategy could confer protection to these cells by improving GJIC and ultimately rescuing retinal vascular and glial cells from HG-induced apoptosis. Therefore, targeting Rab20 overexpression could be useful in improving cell–cell communication in retinal endothelial cells and Müller cells, and preventing neurovascular disruption associated with diabetic retinopathy.
